# The Rate of Physicochemical Incompatibilities, Administration Errors. Factors Correlating with Nurses' Errors

**Published:** 2015

**Authors:** Fanak Fahimi, Aida Sefidani Forough, Sepideh Taghikhani, Leila Saliminejad

**Affiliations:** a *Clinical Pharmacy Department, School of Pharmacy, Shahid Beheshti University of Medical Sciences, Tehran, Iran.*; b *Chronic Respiratory Disease Research Center (CRDRC), NRITLD, Masih Daneshvari Hospital, Tehran, Iran.*; c *Lung Transplantation Research Center, NRITLD, Masih Daneshvari Hospital, Tehran, Iran.*

**Keywords:** Physiochemical incompatibility, Preparation and administration error, Medication error, IV medication, Intravenous administration

## Abstract

Medication errors are commonly encountered in hospital setting. Intravenous medications pose particular risks because of their greater complexity and the multiple steps required in their preparation, administration and monitoring. We aimed to determine the rate of errors during the preparation and administration phase of intravenous medications and the correlation of these errors with the demographics of nurses involved in the process. One hundred patients who were receiving IV medications were monitored by a trained pharmacist. The researcher accompanied the nurses during the preparation and administration process of IV medications. Collected data were compared with the acceptable guidelines. A checklist was filled for each IV medication. Demographic data of the nurses were collected as well. A total of 454 IV medications were recorded. Inappropriate administration rate constituted a large proportion of errors in our study (35.3%). No significant or life threatening drug interaction was recorded during the study. Evaluating the impact of the nurses’ demographic characteristics on the incidence of medication errors showed that there is a direct correlation between nurses’ employment status and the rate of medication errors, while other characteristics did not show a significant impact on the rate of administration errors. Administration errors were significantly higher in temporary 1-year contract group than other groups (*p*-value < 0.0001). Study results show that there should be more vigilance on administration rate of IV medications to prevent negative consequences especially by pharmacists. Optimizing the working conditions of nurses may play a crucial role.

## Introduction

Medication errors are defined as “any preventable event that may cause or lead to an inappropriate medication use or patient harm while in the control of the health care professional, patient or consumer” (1). Estimations indicate that 1-2% of patients admitted to hospitals in the USA are harmed due to medication errors (2). Medication errors can be a major cause of adverse events. One of the most serious adverse events that can occur as a result of medication error is one that involves the intravenous (IV) route of administration. Intravenous administration is the most common route of drug administration in the critical care setting; therefore this population is at high risk for adverse drug events (3). One study estimated about one-half of medication errors occurred in IV preparations and administrations, 1% of which resulted in severe adverse events (4).

Parenteral therapy is one of the various routes of administration especially for drugs which are poorly absorbed via the oral route and it can benefit healthcare providers to gain a rapid response in patients especially during an emergency situation. Nevertheless, inappropriately prepared and/or administered parenteral therapy can result in harm to patients such as thrombus formation, hypersensitivity reactions and infection (5).

Medication administration errors (MAEs) are frequently encountered and are more likely to lead to serious harm and death compared to other types of medication errors (6). To date, most of the scientific studies on medical administration errors have been centered around oral medications or medications administered during regular drug rounds. However, fewer findings about MAEs resulted from IV bolus doses or intermittent infusions have been reported (7).

Intravenous medications create particular risks because of their greater number of steps involved in their preparation, administration and monitoring. Relatively few studies have investigated the incidence of IV medication administration errors, but those conducted confirm the high rate of these errors (6). In hospital setting, polymedication is a common situation in inpatients, especially in elderly patients who are at higher risk of developing adverse reactions resulting from medication errors (8).

Physicochemical incompatibilities between injected drugs are one of the likely errors in hospital settings. Physicochemical incompatibility refers to physical (precipitation, effervescence, changes in color) or chemical (≥10% of degradation of one or more of the components of a preparation within 24 hours) incompatibilities (9). Incompatibilities may be seen between two drugs and also between a drug and a solute, an adjuvant (preservative, buffer, stabilizer and solvent), even a container or a medical device. Unlike interactions which occur in the body, physicochemical interactions arise during the preparation or administration of drugs.

An overview of IV-related medication administration errors during a 5-year period, reported IV medication errors were 73769 cases. These errors mainly resulted from improper concentration and mistakes in calculations (10).

The study by Fahimi *et al. *showed that using inappropriate diluents for preparation IV medication constituted 11.2% of overall errors while the percentage of incompatibility errors was 0.2% (3). In a study by Kalikstad *et al*. 74% of drug co-infusions in neonates had either been incompatible or had not been tested (11).

A previous study showed that risks of medication errors increase when the working hours of nurses are prolonged (12). Also, experienced nurses less likely make medication errors (13).

The objective of this study was to determine the rate of physicochemical incompatibilities of IV medications with both solvents and/or with other IV medications, incorrect concentration, and incorrect rate of administration. Also, we aimed to find out whether any correlation exists between the demographic characteristics of the nurses and the rate of mentioned errors. 

## Experimental

*Method*

This study was carried out in a tertiary care respiratory center, Masih Daneshvari hospital, a university affiliated center, located in Tehran-Iran during a 1-year period. Currently, in this institution, IV medications are mostly prepared by the nurses in the wards and pharmacist involvement is minimal in medication preparation.

One hundred patients regardless of their gender, age or diagnosis who were admitted to different wards of the hospital (medical and surgical ICU, internal, oncology, CCU and post-CCU) were enrolled in the study. The patients were supposed to receive at least one IV medication in order to be included in the study. 

A pharmacist who was trained in observation based medication error research, accompanied nurses during IV drug preparation and administration rounds. All data including patients’ demographic data, name of the responsible nurse, physicians’ name and the name of the IV medication were collected in a questionnaire.

These data were then evaluated based on the medical guidelines from different resources in order to recognize any possible physicochemical incompatibility of each IV medications with diluents and solvents as well as other medications prescribed concomitantly. Physicochemical incompatibility and rate of administration data was extracted from two different references (14, 15).

Drug interaction data were also recorded for each patient. Drug-drug interactions data were obtained from two different references (16, 17).

Direct observation method was used to detect potential errors. The investigator followed the process of preparation and administration without any intervention as long as no serious or life-threatening condition (*e.g*. precipitation of medication) was encountered. In case of life-threatening situations or any error which was likely to cause imminent harm, the observer intervened during the observation process due to ethical issues although it might have changed the results of the study. The final outcomes of the study were categorized in four following concepts:

- Concordance of the physicians' orders with existing medical guidelines and selected references.

- Concordance of the nurses' administration method in terms of the dose, rate and dilution method with the physician's order.

Recognizing potential physicochemical incompatibilities during the preparation phase.

- Observance of drug-drug interactions during preparation of more than one IV drug.

In order to remove between session variations, we applied a correction factor since the number of observations for each medication was different. Correction factor expresses the number of opportunities for errors for each prescribed medication. In this way, we were able to find the rate of errors for each medication under equal conditions.

Nurses' demographic data including age, gender, level of education, work experience and employment status were retrieved from hospitals' nurse office for further analysis. All of the included nurses remained anonymous during and after the study. Needed to mention that enrolled nurses were serving under four different categories of employment status described as 1. Official contracts 2. Three-year contract staff (renewed every 3 years) 3. Intern nurses 4. One-year contract personnel (renewed annually).

Statistical analysis was performed by SPSS 19.0 (SPSS Inc., Chicago, IL). The method of statistical analysis was General Estimating Equation (GEE).

## Results

A total of 454 files for 21 IV medications, related to 100 admitted patients in different wards were evaluated in this study. Thirty two nurses were in charge of preparation and administration of the medications to the enrolled patients.

Among 21 IV medications, the most frequently prescribed drugs were phenytoin (16.2%), clindamycin (12.5%), cefazolin (12.3%), ceftriaxone (10.3%), pantoprazole (5.9%) and meropenem (5.9%).

Drug-drug interactions were detected for only 4 cases (0.9%) including co-administration of pantoprazole and amphotericin B, concomitant use of vancomycin and amphotericin B and co-administration of furosemide and hydrocortisone (2 cases).

No significant or major interactions were seen among the rest of prescribed medications (99.1%). Physiochemical incompatibilities were observed in only 2 (0.44%) evaluated drug charts which were due to clindamaycin- ceftriaxone and vancomycin-dexamethasone combination.

**Figure 1:**
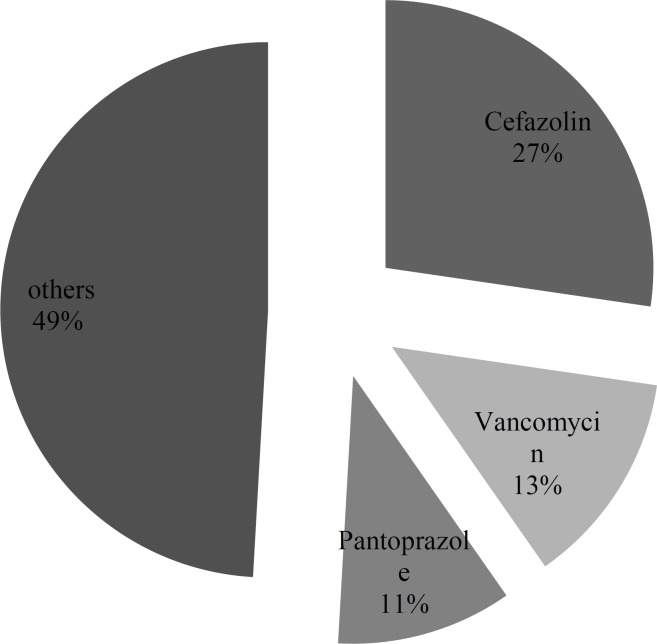
Percentage of inappropriate injection rate before correction factor.

**Figure 2:**
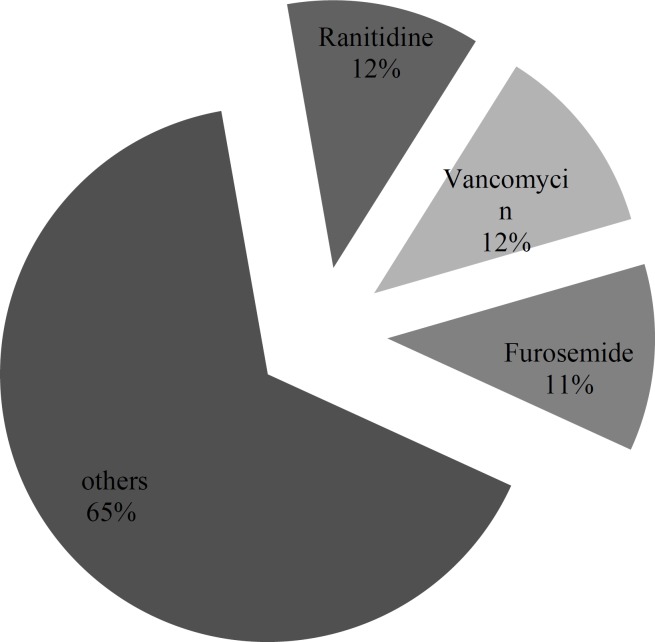
Percentage of inappropriate injection rate after correction factor.

Inappropriate administration rate was the main error found in this study with 161 (35.4%) cases of total 454 administrations. This type of error occurred as a higher rate of administration than the recommended rate by guidelines in all cases. Cefazolin, vancomycin and pantoprazole constituted the largest proportion of administration rate error with 27.3%, 13.0% and 10.6% respectively. However, after applying correction factor ranitidine, vancomycin and furosemide constituted the largest proportion of administration rate error with 11.7, 11.6 and 11.3%, respectively. Figure 1 and Figure 2 demonstrate the inappropriate administration rate for each of the studied IV medications.

Detailed information regarding the incidence of administration rate error for prescribed medications is presented in Table 1.

**Table 1:** Percentage of administration rate error in prescribed IV medications

Name of the IV medication	Number of observations	Number of errors	Rate of errors before applying correction factor (%)	Number of errors after applying correction factor	Rate of errors after applying correction factor (%)
Ranitidine	13	12	7.5	92.3	11.7
Vancomycin	23	21	13.0	91.3	11.6
Furosemide	18	16	10.0	88.9	11.3
Cefazolin	56	44	27.3	78.6	10.0
Ciprofloxacin	11	8	5.0	72.7	9.3
Metoclopramide	7	5	3.1	71.4	9.1
Pantoprazole	27	17	10.6	63.0	8.0
Methylprednisolone	4	2	1.2	50.0	6.4
Metronidazole	6	3	1.9	50.0	6.4
Paracetamol	16	7	4.3	43.8	5.6
Morphine	4	0	0	0	0
Phenytoin	74	11	6.9	14.8	1.9
Piperacillin/tazobactam	9	1	0.6	11.1	1.4
Ceftriaxone	47	5	3.1	10.6	1.3
Clindamycin	57	2	1.2	3.5	0.4
Dexamethasone	13	2	1.2	15.3	1.9
Meropenem	27	1	0.6	3.7	0.5
Hydrocortisone	20	3	1.9	15.0	1.9
Tazobactam	10	1	0.6	10.0	1.3
Imipenem	8	0	0	0	0
Ceftazidime	4	0	0	0	0
Total	454	161	100	786	100

Regarding the demographic data of studied nurses, the male: female ratio was 2:30 with the mean job experience of 7.5 years. All the nurses participated in this study held a bachelor's degree.

In terms of job status, 61.7% of nurses in studied files were official employees while 13.7% were 1-year contract staffs, 2.4% were spending their 2-year period of governmental commitment service and 22.1% were 3-year contract staff (Table 2).

**Table 2:** Nurses’ employment status

Employment Status	Frequency	Valid Percent
Official staff	284	%61.7
3-year contract staff	102	%22.2
1-year Contract staff	63	%13.7
Interns	11	%2.4
Total	460	100.0

Evaluation of the impact of the nurses’ demographic characteristics on the incidence of medication errors showed a significant correlation between employment status and the rate of medication errors. Administration errors were significantly higher in 1 year contract staff than other groups (p-value < 0.0001).

Other characteristics did not show a significant impact on the study outcomes.

## Discussion

Results from our study showed that the administration rate was inappropriate in more than 35% of IV administrations. As an example, it is recommended that vancomycin should be infused in at least 60 minutes; while in 91.3% of vancomycin orders in our study, it was administered faster than the standard pattern.

There can be various factors contributing to such elevated rate of medication errors. First, most of the charts are lacking the administration rate ordered by the physician in charge. Lack of nurses’ knowledge about standard infusion rates can be another influential factor. Additionally, nurses’ heavy work schedule and long working hours may lead to poor personnel commitment even if they are aware of the standard and recommended infusion rates. Since pharmacist involvement is suboptimal during the administration process in our center, it is not possible to prevent most of these medication errors. In a study which assessed the rate and sort of medication errors and their main causes in an emergency room of Imam Khomeini hospital in 2013, most errors occurred were related to incorrect infusion rate (33%) and the staff shortage was recognized as the main contributing factor (18).

In another study which was performed in a teaching hospital in Sydney, Australia with the aim to evaluate the impact of nurses’ experience on submitted errors, wrong infusion rate was again announced as the most frequent error (6).

By optimizing the rate of infusion especially with pharmacist involvement, most of the adverse effects that are associated with the high speed of infusion can be prevented.

Drug incompatibilities are abundantly documented in the literature. A Swiss study (9) made in two different adult intensive care units and a medicine unit showed that 3.4% of drugs combinations used were incompatible.

According to the obtained results from our study, the rate of medication interactions (0.9%) and physiochemical incompatibilities (0.4%) were almost ignorable. This may be due to the non-simultaneous injection of different IV drugs as well as refraining from using same IV lines for different medications.

Taking nurses' crucial role in the process of administration into account, we tried to find any possible relation between the nurses' demographic data as well as their work and educational qualities with the rate of these errors. Results of this study confirmed that, there is not a significant correlation between age, and job experience of the enrolled nurses and the rate of medication errors, while working status (employment condition) shows a significant effect (p-value < 0.0001) on the rate of administration errors. It should be noted that due to having only two males among nurses the analysis on gender was not possible. Detailed analysis showed that errors were significantly higher in nurses who were working with a temporary 1-year contract (Table 2). Lack of job security which brings lower commitment might be considered as the main reason of this notable difference. Hiring well-trained nurses with secure working status and alleviating their heavy work load might assist us to prevent medication errors to some extent. Organizing workshops for nurses and healthcare professionals can be advantageous in this case.

Our study’s results conflict with what has been derived from another study (13) which has found a meaningful relation between the work experience of nurses and better patient care with fewer errors. One explanation would be the more careful attitudes of newly employed staff (*e.g*. interns) towards obeying the exact recorded orders while more experienced staff might be not as accurate as young ones, leading to closure of the gap between experienced and non- experienced nurses.

Detrimental effect of working overtime as a determining factor for increased number of medication errors has been demonstrated by a previous study (19). Hectic working environment especially in emergency department was one of the main factors leading to high incidence of medication errors in one study (20).

Despite the fact that in some cases the correct administration order was already recorded in the patient’s file by physician or pharmacist, it seems that these orders were ignored by the nurses intentionally in order to infuse the medications much faster to save their time. In a survey conducted by Tang *et al*. in 2007 the majority of respondent nurses stated that personal neglect is the most common cause of medication errors by nurses (21). Moreover, a recent review study showed that the fear of punitive actions and reporting process are two factors that make the healthcare providers refrain from reporting medication errors (22). It may be beneficial to investigate the nurses’ attitudes towards medication error and finding out their definition of it since they may be unaware of the potential consequences.

This investigation was subject to some limitations, as the staff in charge of infusions may not obey the recommendations from pharmacist or they may follow the instructions occasionally only when they are under researcher’s supervision. Additionally, the number of shifts for each nurse was not available in this study. So, we were not able to compare the effect of number of shifts as a contributing factor in nurses' performance.

## References

[1] Ross LM, Wallace J, Paton JY (2000). Medication errors in a paediatric teaching hospital in the UK: five years operational experience. Arch. Dis. Child.

[2] Williams DJP (2007). Medication errors. JR. Coll. Physicians Edinb.

[3] Fahimi F, Ariapanah P, Faizi M, Shafaghi B, Namdar R, Ardakani MT (2008). Errors in preparation and administration of intravenous medications in the intensive care unit of a teaching hospital: an observational study. Aust. Crit. Care.

[4] Taxis K, Barber N (2004). Incidence and severity of intravenous drug errors in a German hospital. Eur. J. Cin. Pharmacol.

[5] Bruce J, Wong I (2001). Parenteral drug administration errors by nursing staff on an acute medical admissions ward during day duty. Drug Saf.

[6] Westbrook JI, Rob MI, Woods A, Parry D (2011). Errors in the administration of intravenous medications in hospital and the role of correct procedures and nurse experience. BMJ Qual. Saf.

[7] Han PY, Coombes ID, Green B (2005). Factors predictive of intravenous fluid administration errors in Australian surgical care wards. Qual. Saf. Health Care.

[8] Hohl CM, Dankoff J, Colacone A, Afilalo M (2001). Polypharmacy, adverse drug-related events and potential adverse drug interactions in elderly patients presenting to an emergency department. Ann. Emerg. Med.

[9] Gikic M, Di Paolo ER, Pannatier A, Cotting J (2000). Evaluation of physicochemical incompatibilities during parenteral drug administration in a paediatric intensive care unit. Pharm. World Sci.

[10] Hicks RW, Becker SC (2006). An overview ofi ntravenous-related medication administration errors as reported to MEDMARX, a national medication error-reporting program. J. Infus. Nurs.

[11] Kalikstad B, Skjerdal Å, Hansen TWR (2010). Compatibility of drug infusions in the NICU. Arch. Dis. Child.

[12] Rogers AE, Hwang WT, Scott LD, Aiken LH, Dinges DF (2004). The working hours of hospital staff nurses and patient safety. Health Affair.

[13] Blegen MA, Vaughn TE, Goode CJ (2001). Nurse experience and education: effect on quality of care. J. Nurs. Admin.

[14] Trissel LA (2009). Handbook on Injectable Drugs.

[15] Fahimi F, Ariapanah P (2008). A Quick Guide for Preparation and Administration of Intravenous Medications in Intensive Care Unit.

[16] Drug Interactions Section http://www.uptodate.

[17] Drug Interactions Checker Section.

[18] Ehsani SR, Cheraghi MA, Nejati A, Salari A, Esmaeilpoor AH, Nejad EM (2013). Medication errors of nurses in the emergency department. J. Med. Ethics Hist. Med.

[19] Scott LD, Rogers AE, Hwang WT, Zhang Y (2006). Effects of critical care nurses’ work hours on vigilance and patients’ safety. Am.J. Crit. Care.

[20] Dabaghzadeh F, Rashidian A, Torkamandi H, Alahyari S, Hanafi S, Farsaei S, Javadi MR (2013). Medication errors in an emergency department in a large teaching hospital in Tehran. Iran. J. Pharm. Res.

[21] Tang FI, Sheu SJ, Yu S, Wei IL, Chen CH (2007). Nurses relate the contributing factors involved in medication errors. J. Clin. Nurs.

[22] Mansouri A, Ahmadvand A, Hadjibabaie M, Javadi MR, Khoee SH, Dastan F, Gholami K (2014). A review of medication errors in iran: sources, underreporting reasons and preventive measures. Iran. J. Pharm. Res.

